# Adiponectin suppresses stiffness-dependent, profibrotic activation of lung fibroblasts

**DOI:** 10.1152/ajplung.00037.2024

**Published:** 2024-08-06

**Authors:** Julia Nemeth, Wioletta Skronska-Wasek, Sophie Keppler, Annika Schundner, Alexander Groß, Tanja Schoenberger, Karsten Quast, Karim C. El Kasmi, Clemens Ruppert, Andreas Günther, Manfred Frick

**Affiliations:** ^1^Institute of General Physiology, Ulm University, Ulm, Germany; ^2^Institute of Medical Systems Biology, Ulm University, Ulm, Germany; ^3^Boehringer Ingelheim Pharma GmbH & Co. KG, Biberach, Germany; ^4^Universities of Giessen and Marburg Lung Center (UGMLC), member of the German Center for Lung Research (DZL), Justus-Liebig University Giessen, Giessen, Germany; ^5^Center for Interstitial and Rare Lung Diseases, Justus-Liebig University Giessen, Giessen, Germany

**Keywords:** adiponectin, idiopathic pulmonary fibrosis, mechano-signaling, p38 MAPK, T-cadherin

## Abstract

Idiopathic pulmonary fibrosis (IPF) is a progressive, irreversible respiratory disease with limited therapeutic options. A hallmark of IPF is excessive fibroblast activation and extracellular matrix (ECM) deposition. The resulting increase in tissue stiffness amplifies fibroblast activation and drives disease progression. Dampening stiffness-dependent activation of fibroblasts could slow disease progression. We performed an unbiased, next-generation sequencing (NGS) screen to identify signaling pathways involved in stiffness-dependent lung fibroblast activation. Adipocytokine signaling was downregulated in primary lung fibroblasts (PFs) cultured on stiff matrices. Re-activating adipocytokine signaling with adiponectin suppressed stiffness-dependent activation of human PFs. Adiponectin signaling depended on CDH13 expression and p38 mitogen-activated protein kinase gamma (p38MAPKγ) activation. CDH13 expression and p38MAPKγ activation were strongly reduced in lungs from IPF donors. Our data suggest that adiponectin-signaling via CDH13 and p38MAPKγ activation suppresses profibrotic activation of fibroblasts in the lung. Targeting of the adiponectin signaling cascade may provide therapeutic benefits in IPF.

**NEW & NOTEWORTHY** A hallmark of idiopathic pulmonary fibrosis (IPF) is excessive fibroblast activation and extracellular matrix (ECM) deposition. The resulting increase in tissue stiffness amplifies fibroblast activation and drives disease progression. Dampening stiffness-dependent activation of fibroblasts could slow disease progression. We found that activation of the adipocytokine signaling pathway halts and reverses stiffness-induced, profibrotic fibroblast activation. Specific targeting of this signaling cascade may therefore provide therapeutic benefits in IPF.

## Graphical abstract

**Figure:**
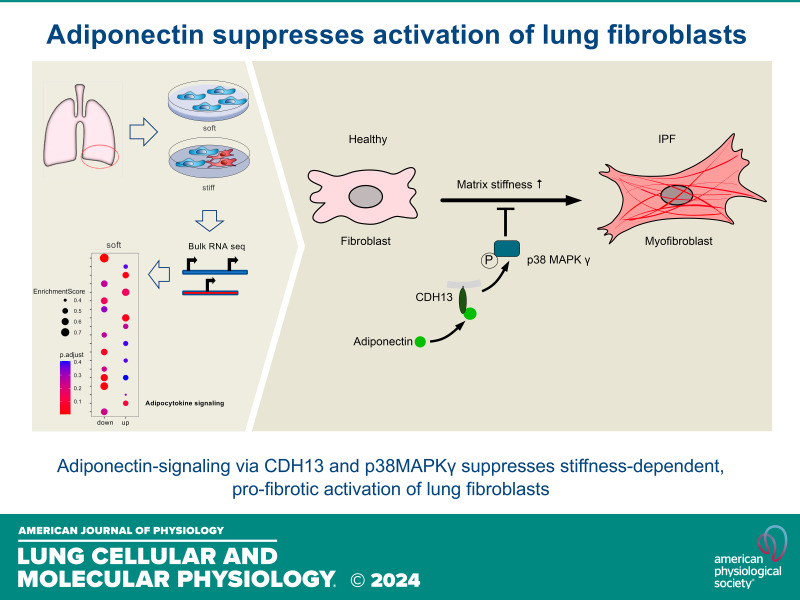

## INTRODUCTION

Idiopathic pulmonary fibrosis (IPF) is a progressive and irreversible respiratory disease with limited therapeutic options (1). A hallmark of IPF is excessive fibroblast activation, differentiation into myofibroblasts and extracellular matrix (ECM) deposition, and cross linking. This results in fibrotic tissue remodeling with increased tissue stiffness, thickened alveolar septae, and impairment of gas exchange, eventually leading to respiratory insufficiency and death (2–4).

In recent years it has become evident that the increasing matrix stiffness is a main driver for fibrosis progression (4, 5). Fibroblasts sense and respond to the increased rigidity of the ECM. This drives a progressive feed-forward loop between increasing matrix stiffness, myofibroblast activation and expression, and cross linking of ECM proteins that perpetuates the fibrotic response (4, 6–11).

Targeting the mechano-transduction pathways underlying the persistent myofibroblast activation has emerged as a potential therapeutic to halt or even reverse fibrosis. Several mechano-transduction pathways that convert mechanical signals into downstream biochemical signals and converge onto transcriptional regulators have been identified (4, 5). In particular, the yes-associated protein (YAP)/transcriptional coactivator with PDZ-binding motif and myocardin-related transcription factor A (MRTF-A) are highly studied mechano-sensing transcriptional regulators implicated in fibroblast function (5, 10, 12). Selective inhibition of such mechano-transduction pathways and myofibroblast activation has been shown to reverse fibrosis (13–15). Identifying additional mechano-signaling pathways could therefore expand the target repertoire and offer novel therapeutic options.

We performed an unbiased, next-generation sequencing (NGS) screen in primary lung fibroblasts to identify signaling pathways affected by increased, profibrotic substrate stiffness. We found that adipocytokine signaling was persistently downregulated in primary lung fibroblasts cultured on stiff matrices. Adipocytokine signaling is generally linked to energy homeostasis and insulin resistance. Recently, however, adiponectin, an adipokine primarily secreted from adipose tissue, has been found to exert antifibrotic activity in various organs including liver, skin, heart, kidney, and the lung (16–18). In the lung, adiponectin protects against paraquat-induced pulmonary fibrosis by decreasing fibroblast activation (19). In line, adiponectin signals are attenuated in lung sections from patients with IPF and systemic sclerosis compared with healthy controls (20). Recent efforts have also attempted to correlate adiponectin levels in serum and broncho-alveolar lavage (BAL) to IPF progression, however, results were not unambiguous (21, 22).

Within this study, we show that adiponectin inhibits substrate stiffness-dependent activation of primary human lung fibroblasts (hPFs) and a recently established pulmonary matrix fibroblast cell line (10-4A) (23). Adiponectin can activate various different signaling pathways via binding to canonical adiponectin receptors (AdipoR1 and R2) or to T-cadherin (CDH13) (16, 24, 25). Here, we report, for the first time, that the antifibrotic effects of adiponectin in lung fibroblasts depend on CDH13 expression and are mediated via activation of p38 mitogen-activated protein kinase gamma (p38MAPKγ). Importantly, we provide evidence that this pathway may be relevant in IPF. CDH13 expression was strongly reduced in lung tissue from IPF donors. Also, activation (phosphorylation) of p38 MAPK isoforms γ/δ was attenuated in the distal lung of patients with IPF. In summary, our data suggest that adiponectin-signaling via CDH13 and p38MAPKγ activation suppresses stiffness-induced, profibrotic activation of lung fibroblasts in the distal lung. This antifibrotic break is downregulated in IPF. Specific targeting of this signaling cascade may provide therapeutic benefits in IPF.

## MATERIALS AND METHODS

Rat adiponectin was obtained from Sigma-Aldrich (26 SRP4903, Steinheim, Germany), rat adiponectin from Abcam (26 ab188451, Cambridge, UK) and human adiponectin from R&D Systems (26 1065-AP-050, Minneapolis, MN), human adiponectin from Sigma-Aldrich (26 SRP4901, Steinheim, Germany). Inhibitors: Dorsomorphin dihydrochloride (26 3093/10), GW 6471 (26 4618/10), SB 706504 (26 5040/10), GSK 1059615 (26 4026/10), Celecoxib (26 3786/10), C 87 (26 5484/10), GW 9662 (26 1508), and BIRB 796 (26 5989) were obtained from R&D Systems. PH 797804 (26 S2726) was obtained from Selleckchem. IMD-0354 (26 I3159) was obtained from Sigma-Aldrich. All other chemicals used for solutions and buffers were obtained from Sigma-Aldrich GmbH (Steinheim, Germany) if not stated otherwise.

### Primary Antibodies Used for Immunofluorescence Staining and Western Blotting

The primary antibodies used for immunofluorescence (IF) staining and Western blotting (WB) were as follows: rb aSMA (IF 1:200, Cat. No. ab5694; Abcam; RRID:AB_2223021), rb aSMA (WB 1:5,000, Cat. No. ab124964; Abcam; RRID:AB_11129103), ms vimentin (IF 1:100, Cat. No. ab8979; Abcam; RRID:AB_306908), rb AdipoR1 (IF 1:100, Cat. No. ab126611, Abcam, RRID:AB_11129655), gt AdipoR2 (IF 1:100, Cat. No. ab77612, Abcam, RRID:AB_1565815), gt Cdh13 (IF 1:200, IHC 1:400, WB 1:1,000, Cat. No. AF3264, R&D Systems, RRID: AB_2077121), rb Phospho-p38 MAPK (Thr180/Tyr182) (WB 1:500, Cat. No. 9211, Cell Signaling, RRID:AB_331641), rb p38 MAPK (Thr180/Tyr182) (WB 1:500, Cat. No. 9212, Cell Signaling, RRID:AB_330713), rb Phospho-p38 MAPK γ/δ (Tyr185, Tyr182) (WB 1:500, Cat. No. PA5-64600, Thermo-Fisher scientific, RRID:AB_2662966), ms HSP90 a/b antibodies (F-8) (1:500, Cat. No. sc-13119; Santa Cruz Biotechnology, Dallas, TX, RRID:AB_675659), and ms GAPDH (1:1,000, Cat. No. sc-365062, Santa Cruz Biotechnology, Dallas, TX, RRID:AB_10847862).

### Secondary Antibodies Used for IF

The secondary antibodies used for IF were as follows: Alexa FluorR 488 goat anti-chicken (1:300, Cat. No. A11039; Thermo Fisher Scientific, RRID:AB_142924); Alexa FluorR 647 goat anti-chicken (1:300, Cat. No. A-21449; Thermo Fisher Scientific, RRID:AB_2535866); Alexa FluorR 568 goat anti-rabbit (1:300, Cat. No. A11011; Thermo Fisher Scientific, RRID:AB_143157); and Alexa FluorR 488 goat anti-mouse (1:300, Cat. No. A11029; Thermo Fisher Scientific, RRID:AB_138404).

### Secondary Antibodies Used for WB

The secondary antibodies used for WB were as follows: IRDye R 800CW donkey anti-rabbit (1:20,000, Cat. No. 926-32213, RRID:AB_621848), IRDyeR 680RD donkey anti-mouse (1:20,000, Cat. No. 926-68072, RRID:AB_10953628), IRDye R 800CW donkey anti-mouse (1:20,000, Cat. No. 926-32212, RRID:AB_621847), IRDyeR 680RD donkey anti-rabbit (1:20,000, Cat. No. 926-68073, RRID:AB_10954442).

### Cell Isolation and Cell Culture

Primary lung fibroblasts were isolated from 12- to 14-wk-old male Sprague-Dawley rats according to the method of Jansing et al. (26) with minor modifications as previously described (23). In short, rats were anesthetized with ketamine (10%) and xylazine (2%) and injected with heparin (400 IU/kg). Lungs were perfused, removed, washed with BSS-A supplemented with EGTA, BSS-A w/o EGTA, and BSS-B solution. The tissue was incubated with 0.5 mg/mL elastase (Elastin Products Co, Owensville, MO) and 0.05 mg/mL trypsin at 37°C for 30 min. Then, 2 mg/mL DNase were added, the enzymatic reaction was stopped by FCS, and the digested tissue was filtered through gauze and nylon meshes (mesh sizes: 100, 70, and 40 μm). For purification of primary fibroblasts, cell suspensions were depleted of leucocytes using anti-CD45 MicroBeads (Miltenyi Biotec, Gladbach, Germany) before fibroblasts were isolated using anti-CD90.1 MicroBeads (Miltenyi Biotec, Gladbach, Germany) according to manufacturer’s instructions. Cells were seeded on polydimethylsiloxane (PDMS) gels or plastic substrate in MucilAir culture medium containing 25.6 µg/mL of gentamicin at a density of 1 to 5 × 10^5^ cells/cm^2^. Cells were cultured at 37°C, 5% CO_2_, and 95% humidity for up to 14 days. Culture media were changed every two days, with adiponectin being added freshly to the medium in the corresponding experiments.

10-4A cells (23) were maintained in the chemically defined, standardized, cell culture medium MucilAir (Epithelix, Genève, Switzerland) containing 25.6 µg/mL of gentamicin (Thermo Fisher Scientific) at 37°C, 5% CO_2_, and 95% humidity. Cells were detached upon reaching 80% confluence using TrypLE (Thermo Fisher Scientific), centrifuged, resuspended in cell culture medium ± 2 µg/mL adiponectin, and seeded on PDMS gels or plastic dishes at a density of 0.5 × 10^3^ to 40 × 10^3^ cells/cm^2^. To obtain a mixture of the different adiponectin complexes that resemble the low-, mid-, and high-molecular adiponectin complexes found in human plasma, HEK-cell derived (rat: Abcam, human: R&D) and *Escherichia coli* (rat and human: both Sigma) derived adiponectin were mixed in a 1:1 ratio. Culture media were changed every two days, with adiponectin being added freshly to the medium in the corresponding experiments. Cells from *passage 11* to *25* were used for all experiments.

### Human Biospecimens

Human biospecimens from patients with IPF undergoing lung transplantation and non-IPF donors were collected in frame of the European IPF registry (euIPFreg) and provided by the UGMLC Giessen Biobank, member of the DZL platform biobanking. *n* = 5 for formalin-fixed, paraffin-embedded (FFPE) tissue and *n* = 8 for snap-frozen tissue. All IPF diagnoses were made according to the American Thoracic Society (ATS)/European Respiratory Society (ERS) consensus criteria (27), and a usual interstitial pneumonia (UIP) pattern was proven in all patients with IPF.

Human pulmonary fibroblasts from healthy donors were obtained from Promocell (Cat. No. C-12360, Promocell, Heidelberg, Germany). Culture conditions were the same as described for 10-4A cells. Age, sex, and ethnicity of donors are listed in Table 1. The sex of the individual donors is represented within the analyzed data. Male donors are encoded by open circles, female donors are encoded by full circles.

**Table 1.** Age, sex, and ethnicity of donors

Lot	Age, yr	Sex	Race
3080702	75	Male	White
407Z037	61	Female	White
419Z020.2	79	Female	White
433Z024	44	Female	White
446Z031	57	Female	White
474Z024.2	73	Male	White
474Z031.2	56	Male	White
480Z007	74	Female	White
484Z018.1	71	Male	White

### PDMS Gel Preparation and Coating of Cell Culture Dishes

PDMS (3 kPa) gels were prepared with the Sylgard 527 Silicon Dielectric Gel Kit (Dow Europe GmbH, Wiesbaden, Germany) as previously described (28) with minor modifications (23). Component A and B were thoroughly mixed in a 1:1 ratio and added to 24-well culture plates (Sarstedt, Nümbrecht, Germany) or ibiTreat μSlide 8 well (ibidi GMBH, Gräfelfing, Germany) respectively. Culture containers were kept under vacuum for 2 h to remove potential air inclusions and then incubated at room temperature for 48 h for polymerization. Fully polymerized PDMS gels were sterilized in a UV Crosslinker (GE Healthcare Europe GmbH, Freiburg, Germany) for 30 min and then coated with a 0.01% wt/vol polydopamine solution [50 mM Tris-HCl, pH = 8.5, 0.01% (wt/vol) dopamine hydrochloride] for 1 h and a 38 µg/mL rat tail collagen I solution (Advanced BioMatrix Inc., San Diego, CA), diluted in Dulbecco PBS (Biochrom, Berlin, Germany; pH = 7.4) over night at 37°C, respectively.

Identical coating conditions were used for culture plastic dishes w/o PDMS to ensure comparability.

For testing of different substrates stiffnesses on cell behavior, CytoSoft PDMS substrates (Advanced BioMatrix, Inc. San Diego, CA) with a stiffness of 0.2, 0.5, 2, 8, 16, 32, and 64 kPa were used.

### RNA Isolation, cDNA Synthesis, and qPCR

RNA was isolated using the my-Budget RNA Mini Kit (Bio-Budget Technologies GmbH, Krefeld, Germany) in combination with the RNase free DNase Set (QIAGEN GmbH, Hilden, Germany). RNA (400 ng) was reverse transcribed into cDNA by using the SuperScript VILO cDNA Synthesis Kit (Thermo Fisher Scientific).

Prior to qPCR, the cDNA was diluted in a 1:3 ratio with DEPC-treated H_2_O (Carl Roth, Karlsruhe, Germany). Amplification was performed on a StepOnePlus qPCR cycler (Applied Biosystems, Foster City, CA) using EvaGreen QPCR Mix II (Bio-Budget Technologies).

The following QuantiTect Primer assays (QIAGEN GmbH, Hilden Germany) were used: Rn_Acta2_1_SG (QT01615901), Hs_ACTA2_1_SG (QT00088102), Rn_Col1a1_1_SG (QT01081059), Hs_COL1A1_1_SG (QT00037793), Rn_Ctgf_1_SG (QT00182021), Hs_CTGF_1_SG (QT00052899), Rn_Tagln_1_SG (QT00188769), Hs_TAGLN_1_SG (QT00072247), Rn_Adipor1_1_SG (QT00442365), Hs_ADIPOR1_1_SG (QT00002352), Rn_Adipor2_2_SG (QT01595580), Hs_ADIPOR2_1_SG (QT00058716), Rn_Cdh13 (Biorad, 10025636), Hs_CDH13_1_SG (QT00067242), and Rn_Hmbs_1_SG (QT00179123), Hs_HMBS_1_SG (QT00014462). The relative quantification of mRNA expression was performed according to the method of Pfaffl (27, 29).

### Illumina Library Preparation and Sequencing

The Sequencing library preparation has been done as described previously (23). Total RNA (200 ng) was used for library preparation with the TruSeq RNA Sample Prep Kit v2-Set B (RS-122-2002, Illumina Inc, San Diego, CA) producing a 275 bp fragment including adapters in average size. Finally, eight individual libraries were normalized and pooled together using the adapter indices supplied by the manufacturer. Pooled libraries have then been clustered on the cBot Instrument from Illumina using the TruSeq SR Cluster Kit v3—cBot—HS (GD-401–3001, Illumina Inc, San Diego, CA). Sequencing was then performed as 50 bp, single reads, and 7 bases index read on an Illumina HiSeq2000 instrument using the TruSeq SBS Kit HS-v3 (50-cycle) (FC-401-3002, Illumina Inc, San Diego, CA). Sequencing data have been deposited in GEO data set GSE199753.

### mRNA-Seq Bioinformatics Pipeline

Raw read counts obtained by sequencing were variance stabilizing transformed (30) for quality control where a visual inspection based on principal component analysis identified a sample as outlier. This sample was removed from further analysis. Based on the raw counts, likelihood-ratio tests (30) were carried out identifying differentially expressed genes across days within both substrates and a day-by-day comparison between the two substrates. Clusters were proposed by taking model coefficients from the models used in the tests, subtraction of the gene-wise minimums, division by the gene-wise maximums, weighting by false discovery rates so that lowly significant genes should tend to show constant values, and scaling by 100 to allow for distinct days to be distinguished in k-means clustering.

### Pathway Analysis

Gene expression data were further analyzed using gene set enrichment analysis (GSEA) (31). Genes with a false discovery rate (FDR) < 0.01 were extracted from the respective clusters and further analyzed. The Kyoto Encyclopedia of Genes and Genomes (KEGG) pathway database (c2.cp.kegg.v.7.4.symbols.gmt) was used for pathway analysis. The Rat_ENSEMBL_Gene_ID_Human_Orthologs_MSigDB.v7.4.chip platform was used for annotation. Enrichment statistics were calculated with the parameter weighted: *P* = 1, as metric for gene rankings the signal to noise ratio was used.

### Western Blotting

Snap-frozen human tissue was grinded with a mortar and pestle. Liquid nitrogen was added to keep the tissue pieces frozen. Tissue powder (10–50 mg) was dissolved in 500 µL of T-Per buffer (Thermo, Cat. No. 78510) containing Pierce Protease and Phosphatase Inhibitor Mini Tablets (Thermo, Cat. No. A32959). The tissue lysate was sonicated for 1 min (Sonifier 250, Branson Ultrasonics Corporation, Danbury, CT) and incubated for 30 min on ice. The lysate was centrifuged for 20 min at 12,000 relative centrifugal force (rcf) (4°C) and the supernatant was transferred into a new tube.

Cells were washed twice with PBS, collected with RIPA buffer (Sigma-Aldrich) and sonicated prior loading on gels.

The protein concentration was determined by Pierce bicinchoninic acid (BCA) Protein Assay (Thermo Fisher Scientific). Protein Loading Buffer and NuPAGE reducing agent (Thermo Fisher Scientific) were added in a 1:5 and 1:10 ratio, respectively. Samples were incubated at 70°C for 10 min, separated by SDS-PAGE and blotted on a nitrocellulose membrane. Primary antibodies and the respective secondary antibodies were incubated for 1 h at room temperature. Membranes were analyzed with the Odyssey Fc Imaging System (LI-COR Biosciences).

AdipoR1 was analyzed by performing native protein blots. Therefore, the Native Page Novex Bis-Tris gel system (Thermo Fisher Scientific) was used. Samples were prepared by washing the cells with PBS and addition of 4x NativePAGE Sample Buffer diluted in deionized water without any further detergents. Samples were separated by using a NativePAGE 4%–16% Novex Bis-Tris Gel (Thermo Fisher Scientific) and blotted on a PVDF-membrane. Primary antibodies were incubated overnight at 4°C and secondary antibodies were incubated for 1 h at room temperature. Membranes were analyzed with the Odyssey Fc Imaging System (LI-COR Biosciences).

### Dot Blot

Samples were harvested in 4x NativePAGE Sample Buffer diluted in deionized water. For HSP90, the respective amount of sample was additionally sonicated and denatured by adding 10x NuPAGE reducing agent, followed by an incubation at 70°C for 10 min. For the dot blot, a nitrocellulose membrane was pre-soaked in Dulbecco’s phosphate-buffered saline (DPBS). The Dot Blot apparatus was assembled under vacuum to avoid leakage. Afterward, the vacuum was removed and a minimal volume of 50 μL/sample was loaded into the respective wells. For blotting, vacuum was applied until the whole sample was pulled through the membrane. The remaining steps are identical as described in *Western Blotting*.

### siRNA Experiments

10-4A wildtype (WT) cells were seeded in 24-well plates at a cell density of 5 × 10^2^ cells/cm^2^. Four days after seeding, the cells were transfected with 50 pmol of the respective Silencer Select siRNA (Thermo Fisher Scientific) using the siTran transfection reagent (Origene) according to the manufacturer’s instruction. After 48 h, samples were used for further experiments (RNA-isolation or WB). The following siRNA assays (all obtained from Thermo Fisher Scientific) were used: rat AdipoR1 (Assay ID: s144017), rat AdipoR2 (Assay ID: Assay ID: 281929), rat Cdh13 (Assay ID: s140447), human ADIPOR1 (Assay ID: s27410), human ADIPOR2 (Assay ID: Assay ID: 35893), and human CDH13 (Assay ID: s2806). As negative control, the Silencer Select nt siRNA (26 AM4621, Thermo Fisher Scientific) was used.

### Immunofluorescence of Lung Tissue

Formalin-fixed and paraffin embedded human lung slices (5 μm) from healthy and IPF donors were obtained from the Gießen Biobank [Universities of Gießen and Marburg Lung Center (UGMLC)]. Deparaffinization of the slices was performed in 100% Xylol (2×, 10 min), followed by rehydration in a descending ethanol series (100%, 90%, 80%, 70%, 50%, each step for 2 min). Slices were pretreated for 30 min in Citrate buffer (pH = 6) at 98°C or 30 min in Tris-EDTA buffer (pH = 9) at 98°C. For blocking and antibody dilution, the ready to use Blocking Solution (26 ZUC007, Zytomed, Bargteheide, Germany) and antibody diluent (26 ZUC025, Zytomed) was used. The blocking solution was incubated for 15 min, and primary and secondary antibodies for 1 h at room temperature. For staining of the nuclei, cells were incubated for 5 min with 4 ng/mL Hoechst 33342 (Invitrogen, Carlsbad, CA) diluted in DPBS. Images were taken on an inverted confocal microscope (Leica TCS SP5, Leica, Germany) using a HCX PL APO CS 20 × 1.25 air objective and HCX PL APO CS 40 × 1.25 oil objective and the corresponding software Leica Application suite (Leica, Wetzlar/Mannheim Germany).

### Immunohistochemistry of Lung Tissue

Human FFPE lung samples were cut into 3 µm sections. Hematoxylin and eosin (H&E) staining was performed according to standard protocols using the Leica ST5020 Multistainer (Leica Biosystems Nussloch GmbH, Nussloch, Germany). Immunohistochemical staining was carried out on the automated Leica Bond RX platform (Leica Biosystems, Melbourne, Australia) using a polyclonal goat anti-human Cadherin-13 antibody (1:400; AF3264, R&D) after heat-induced epitope retrieval with Bond Epitope Retrieval Solution 1 (ER1, Leica Biosystems, Newcastle, UK) for 30 min. After incubation with a biotinylated secondary antibody (rabbit anti-goat IgG; 1:200; BA-5000, Vectors Labs), bound antibodies were visualized using the Bond Intense R Detection System (Leica Biosystems, Newcastle, UK). Tissues were imaged using a Zeiss Imager.A2 microscope (Carl Zeiss Microscopy GmbH, Germany).

### Microplate BFP Reporter Cell Assay

10-4A^BFP^ cells were seeded to collagen-coated black 96-well plates (26 137101, Thermo Fisher Scientific). After adherence of the cells (∼6 h), the respective inhibitors were added. The medium was changed every 2 days. The BFP signal of the cells was measured at *day 7* postseeding. For normalization, cells were stained with 2.5 µg/mL of Calcein AM (Thermo Fisher Scientific) for 30 min at 37°C, 5% CO_2_ in bath solution (in mM: 140 NaCl, 5 KCl, 1 MgCl_2_, 2 CaCl_2_, 5 glucose, and 10 HEPES; pH = 7.4). After staining, cells were washed three times with PBS w/o Ca^2+^/Mg^2+^ and maintained in 200 µL of bath solution during analysis of the respective BFP and Calcein signal with the plate reader (Tecan, Salzburg, Austria). The excitation and emission wavelengths were as follows: BFP 385 nm/445 nm, Calcein 485 nm/535 nm. For both signals, the background was subtracted and the BFP signal was normalized to the respective Calcein signal.

### Cloning and Expression of p38 MAPK Phosphomimetics

To create phosphomimetic mutants of the respective p38 MAPK subunits, the threonine and tyrosine (at position 183 and 185 for the γ subunit and 180 and 182 for the α and δ subunit) were replaced by glutamic acid using the In-Fusion HD Cloning Plus kit (Takara Bio Inc., Kusatu, Japan). The respective DNA sequences from MAPK12 (p38 γ), MAPK13 (p38 δ), and MAPK14 (p38 α) were obtained from genomic DNA isolated from hPFs using the DNeasy Blood & Tissue kit (Qiagen GmbH, Hilden, Germany) and afterward PCR amplified. The respective MAPK sequences and a T2A-BFP-NLS sequence was integrated into the p-EYFP-N1 Vector backbone (Addgene, Cat 6006-1) using the In-Fusion HD Cloning Plus kit according to manufacturer’s instructions. On this occasion, the EYFP was replaced by the T2A-BFP-NLS sequence.

To mutate the different MAPK subunits, the following primers were used:

MAPK12 human (hs_MAPK12_T183E-Y185E):

fw: GATGGAAGGGGAGGTGGTGACCCGGTGGTACC

rev: ACCTCCCCTTCCATCTCACTGTCTGCCTGCC

MAPK13 human (hs_MAPK13_T180E-Y182E):

fw: GATGGAAGGCGAGGTGGTGACCCGCTGGTACC

rev: ACCTCGCCTTCCATCTCGGCGTCTGCATGTCG

MAPK14 human (hs_MAPK14_T180E-Y182E):

fw: AATGGAAGGCGAGGTGGCCACTAGGTGGTACAGG

rev: ACCTCGCCTTCCATTTCATCATCTGTGTGCCGAGC

hPFs were transfected with the respective p38 MAPK (pMAPK) plasmids using the FuGENE HD Transfection Reagent (Promega, Madison, WI) according to the manufacturer’s instructions. Cells were transfected seven days after seeding with 5 µg of the respective plasmid DNA and 0.75 µL of FuGENE HD Transfection Reagent. Two days after transfection, samples were harvested for WB or qPCR. Transfection efficiency was validated on a test basis (nuclear BFP signal) and was estimated to be between 50% and 65%.

### Statistical Analysis

GraphPad Prism7 software (GraphPad, La Jolla, CA) was used for statistical analysis and data representation. Respective tests are given within the figure legend. Data are represented as means ± SD unless stated otherwise. Statistical significance was determined using the nonparametric Mann–Whitney *U* test for comparison of two independent samples at the same time point and Kruskal–Wallis Test with post hoc Dunn’s test for multiple comparison tests. The number of experiments (*n*) indicates individual animals for primary fibroblasts and cells from varying passages for immortalized fibroblasts. Data were considered significant if the *P* value was <0.05. *P* values < 0.05 are indicated in graphs.

### Study Approvals

The study protocol for human biospecimen was approved by the Ethics Committee of the Justus-Liebig-University School of Medicine (No. 111/08: eurIPFreg and 58/15: UGMLC Giessen Biobank), and informed consent was obtained in written form from each subject.

## RESULTS

### Adipocytokine Signaling Is Persistently Downregulated in Primary Lung Fibroblasts Cultured on Stiff Substrate

**Figure 1.**
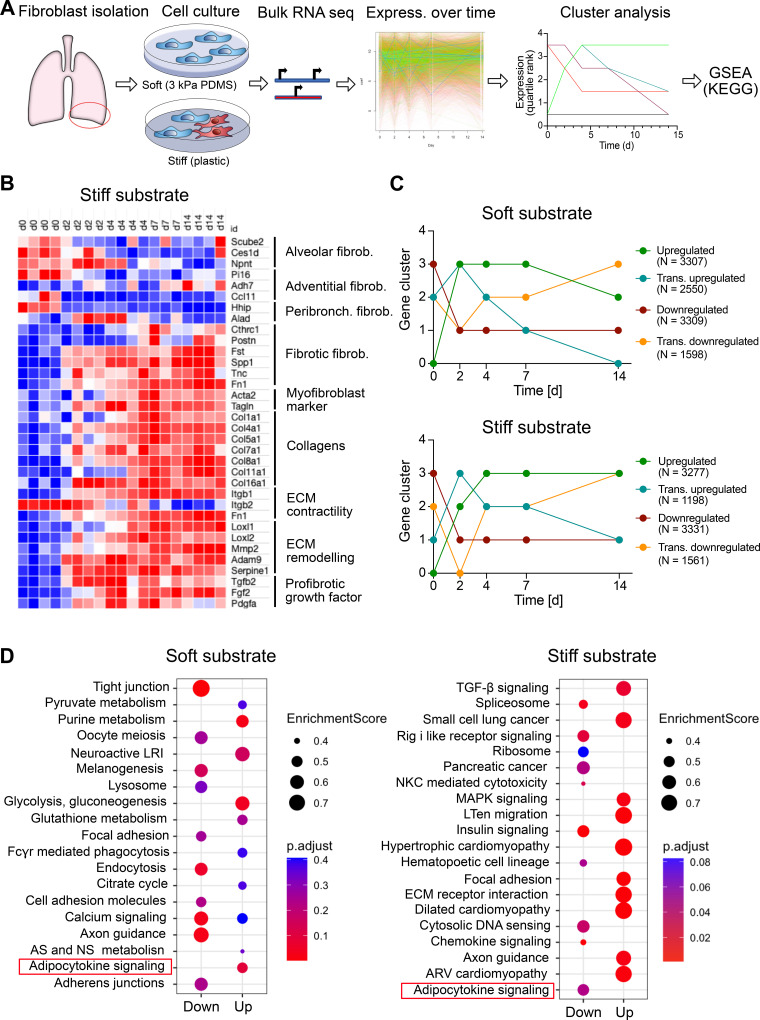
Adipocytokine signaling is downregulated in primary lung fibroblasts cultured on stiff substrate. *A*: schematic representation of primary cell isolation from lung tissue, cell culture conditions, and bulk RNA sequencing (RNA-Seq) analysis workflow. *B*: heat map of scaled gene expression in primary fibroblasts. Expression of representative genes for the fibroblast subtypes (33) and profibrotic marker clusters indicated on the *right* is depicted for cells directly after cell isolation (*day 0*) or in cells cultured on plastic for two (*day 2*), four (day 4), seven (*day 7*) or 14 (*day 14*) days postseeding, respectively. VST counts were used for heat-map presentation. Color coding is adjusted to the minimum and maximum value of gene expression. Red indicates the maximum value and blue the minimum value. *C*: cluster assignment (k-means) of genes expressed in cells maintained on either soft [3 kPa polydimethylsiloxane (PDMS)] or stiff (plastic) substrate over a 14-day time course. Genes were assigned to clusters of either permanently or transiently up- or downregulated genes over the entire time-course. Data were obtained from four individual cell isolations for all conditions. *D*: top ten up- and downregulated pathways in cells seeded on either soft or stiff substrate. For pathway analysis, gene set enrichment analysis (GSEA) in combination with the Kyoto Encyclopedia of Genes and Genomes (KEGG) database was used. Data were obtained from four individual cell isolations for all conditions.

We initially sought to identify signaling pathways altered in response to increased substrate stiffness. To this end, we performed an unbiased analysis of longitudinal changes in gene expression in primary rat lung fibroblasts grown on either physiologically soft [3 kPa PDMS (4, 13, 32)] or stiff (plastic, GPa) substrates. Cells were maintained on the different substrates for 14 days and samples were collected at various time points. Gene expression was then analyzed by bulk RNA sequencing (RNA-seq) (Fig. 1*A*). Examining the expression of gene signatures for different, recently described, subsets of collagen producing lung cells (33) revealed that the isolated primary cells represented alveolar fibroblasts. These are presumed progenitors of fibrotic (CTHRC1+) fibroblasts (33). The analysis of longitudinal changes in the expression of genes linked to profibrotic fibroblast activation confirmed time-dependent, profibrotic activation of the primary fibroblasts, differentiation into myofibroblasts and upregulation of genes linked to increased ECM production, contractility, and remodeling (Fig. 1*B* and Supplemental Fig. S1) (10, 33). Overall, a total of 10,755 and 9,367 genes were differentially expressed over the 14-day period in fibroblasts grown on either soft or stiff matrices, respectively (FDR < 0.01). To identify genes with similar regulation patterns, we performed k-means clustering analysis on the differentially expressed genes. We identified clusters of genes that were either transiently or persistently up- or downregulated on both substrates (Fig. 1*C*). We performed combined gene-set enrichment (GSEA) and KEGG pathway analysis on the permanently up- or downregulated clusters. These likely reflect persistent adaptations to substrate stiffness (Fig. 1*D*). KEGG analysis of the respective gene clusters identified in fibroblasts grown on stiff substrate revealed various gene sets known to be differentially regulated in fibrosis and in response to stiff matrices (e.g., transforming growth factor (TGF)-β signaling pathway, regulation of actin cytoskeleton, and ECM receptor interaction).

In addition, we found that the adipocytokine signaling pathway was significantly and persistently downregulated in primary rat lung fibroblasts cultured on stiff substrate. Interestingly, the adipocytokine signaling pathway was upregulated in primary rat lung fibroblasts grown on soft substrate (Fig. 1*D*). Further analysis revealed that adiponectin was the most significantly deregulated (downregulated) adipokine when comparing transcripts in fibroblast grown on plastic with fibroblast grown on PDMS for 14 days (Supplemental Fig. S2). Interestingly, leptin, often associated with opposite effects of adiponectin, was significantly upregulated in fibroblast grown on plastic compared with fibroblast grown on PDMS for 14 days (Supplemental Fig. S2).

### Adiponectin Suppresses Stiffness-Induced, Profibrotic Fibroblast Activation

**Figure 2.**
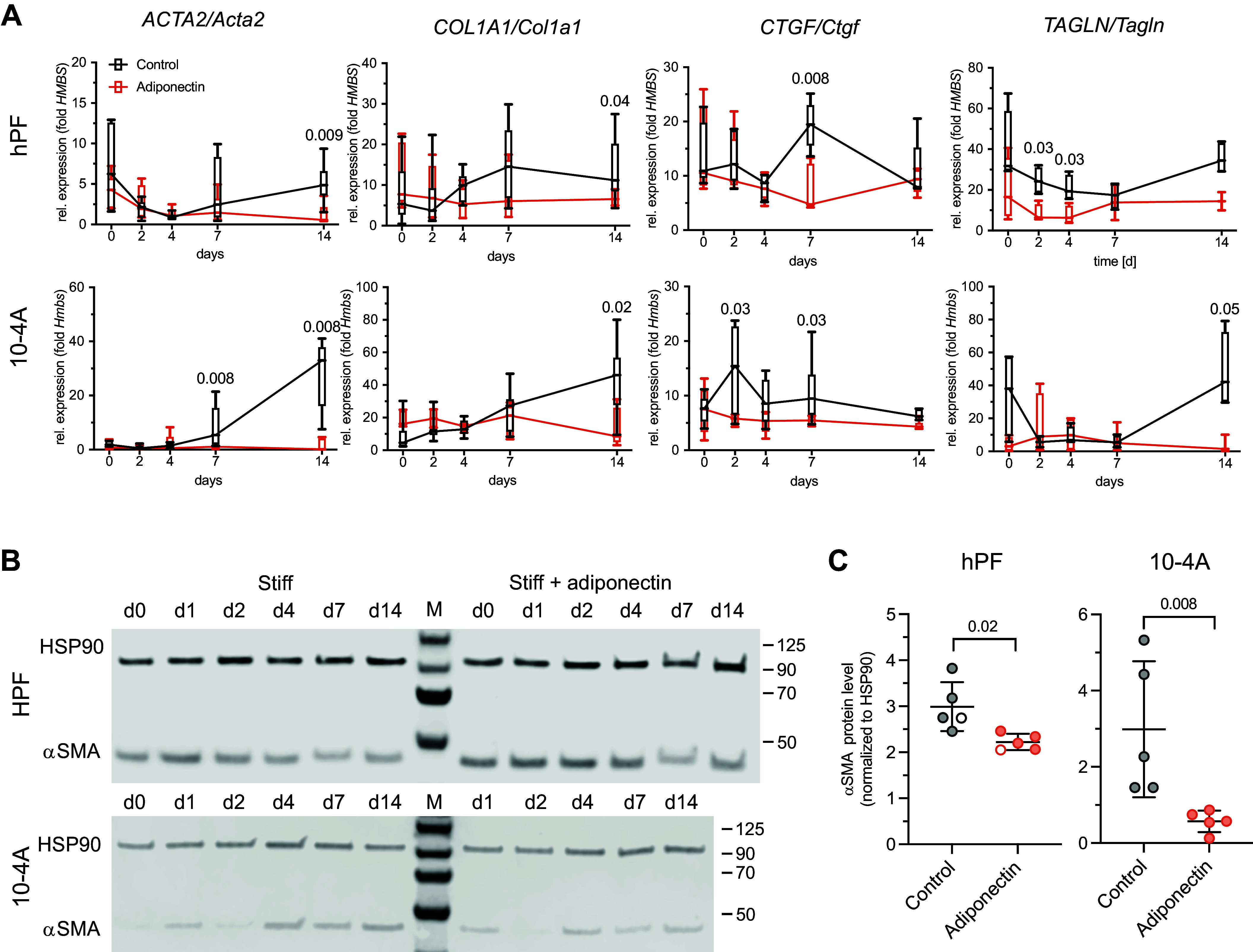
Adiponectin suppresses stiffness-induced, profibrotic fibroblast activation. *A*: real-time RT-PCR analysis of *ACTA2*/*Acta2*, *COL1A1*/*Col1a1*, and *CTGF*/*Ctgf* transcripts in primary human lung fibroblasts (hPFs) and 10-4A cells grown on stiff (plastic) substrate for up to 14 days. Cells were maintained in the absence (control) or presence of 2 µg/mL human or rat adiponectin, respectively. Data are expressed as fold expression of housekeeping gene *HMBS*/*hmbs*, *n* = 5–10 individual isolations (hPF) or passages (10-4A). *B*: Western blots for α-smooth muscle actin (αSMA) expressed in hPF or 10-4A cells grown on stiff (plastic) substrate for up to 14 days. Cells were maintained in the absence (control) or presence of 2 µg/mL rat or human adiponectin, respectively. HSP90 was used as loading control and for protein normalization. *C*: densiometric analysis of αSMA protein expression at *day 7*. All data were obtained from five independent experiments. ○ encode male donors. Significance was assessed by nonparametric Mann–Whitney *U* test.

Adiponectin is one of the main adipocytokines and has been found to exert antifibrotic activity in various organs including liver, skin, heart, kidney, and the lung (16–18). We therefore asked whether physiological levels of adiponectin reduce stiffness-induced fibroblast activation. Levels of adiponectin in human plasma have been reported in the low (1–20) µg/mL range (34) and even lower in lung tissue (22). Treatment of primary human pulmonary fibroblasts (hPFs) or a recently described, fibrosis-sensitive, fibroblast cell line (10-4A) (23) with 2 µg/mL of adiponectin significantly reduced stiffness-induced expression of profibrotic markers *ACTA2/Acta2, COL1A1/Col1a1*, *CTGF/Ctgf and TAGLN/Tagln*, respectively (Fig. 2*A*). This effect was also observed on the protein level. Adiponectin treatment significantly reduced α-smooth muscle actin (αSMA) expression in hPFs and 10-4A cells (Fig. 2, *B* and *C*). Suppression of profibrotic gene expression in fibroblasts has been demonstrated via several mechanisms including proliferation (35). We therefore tested whether adiponectin treatment affects lung fibroblast proliferation. Adiponectin did not change expression of proliferation genes in 10-4A cells and hPFs (Supplemental Fig. S3). In summary, these results suggest a direct antifibrotic effect of adiponectin on stiffness-mediated activation of lung fibroblasts.

**Figure 3.**
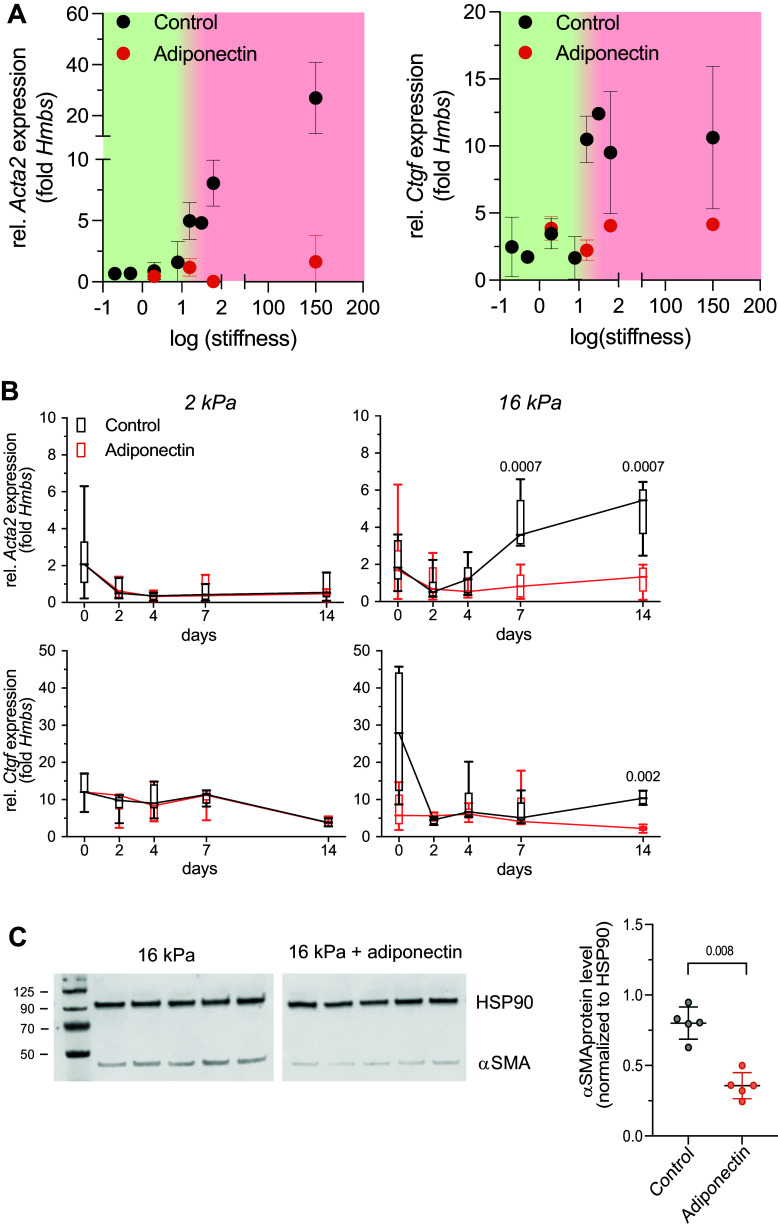
Adiponectin inhibits mechano-signaling induced fibroblast activation across the range of matrix stiffnesses reported from patients with idiopathic pulmonary fibrosis (IPF). *A*: real-time RT-PCR analysis of Acta2 (*left*) and Ctgf expression in 10-4A cells grown on polydimethylsiloxane (PDMS) substrates mimicking the stiffness of healthy (green background) and diseased (red background) lung tissue and plastic (GPa) for 14 days in the absence (control) or presence of 2 µg/mL of rat adiponectin. *n* = 4–7 individual experiments. *B*: real-time RT-PCR analysis of *Acta2* and *Ctgf* transcripts in 10-4A cells grown on PDMS substrate representing matrix stiffness of healthy (2 kPa) or fibrotic (16 kPa) lung. Cells were maintained in the absence (control) or presence of 2 µg/mL of human or rat adiponectin, respectively. Data are expressed as fold expression of housekeeping gene *Hmbs, n* = 5–7 individual experiments. *C, left*: Western blots for αSMA expressed 10-4A cells grown on 16 kPa PDMS substrate for 7 days. Cells were maintained in the absence (control) or presence of 2 µg/mL of rat adiponectin, respectively. HSP90 was used as loading control and for protein normalization. *C*, *right*: densiometric analysis of α-smooth muscle actin (αSMA) protein expression from blots on the left. Significance was assessed by nonparametric Mann–Whitney *U* test.

The initial screen and subsequent confirmation of the effect of adiponectin on stiffness-induced profibrotic activation of fibroblasts was performed in cells grown on tissue culture plastic. Tissue culture plastic, however, represents an unphysiologically high substrate stiffness (GPa). Normal lung tissue has a stiffness in the range of 0.5–5 kPa and fibrotic lung tissue is in the range of 15–100 kPa (4). We therefore tested whether adiponectin also inhibits fibroblast activation under more (patho)physiological conditions. We cultured lung fibroblasts for 14 days on substrates of different stiffnesses mimicking the stiffness of healthy and diseased lung tissue (0.5–64 kPa). Stiffness-dependent fibroblast activation was observed at a Young’s modulus of around 16 kPa (Fig. 3*A*) with similar kinetics to the effects observed in cells maintained on plastic (Fig. 3*B*). This is in line with the increased matrix stiffness levels found in lungs from patients with IPF (7). Adiponectin inhibited the stiffness-induced activation of lung fibroblasts across the entire range of matrix stiffnesses reported from patients with IPF, inhibiting expression of profibrotic markers on the transcript (Fig. 3, *A* and *B*) and protein level (Fig. 3*C*). Adiponectin had no effect on the expression of profibrotic markers in cells maintained at soft substrates mimicking healthy lung tissue (Fig. 3, *A* and *B*), confirming the antifibrotic effect of adiponectin in a physiologically relevant setting.

### The Antifibrotic Effects of Adiponectin Depend on CDH13 Expression in Lung Fibroblasts

**Figure 4.**
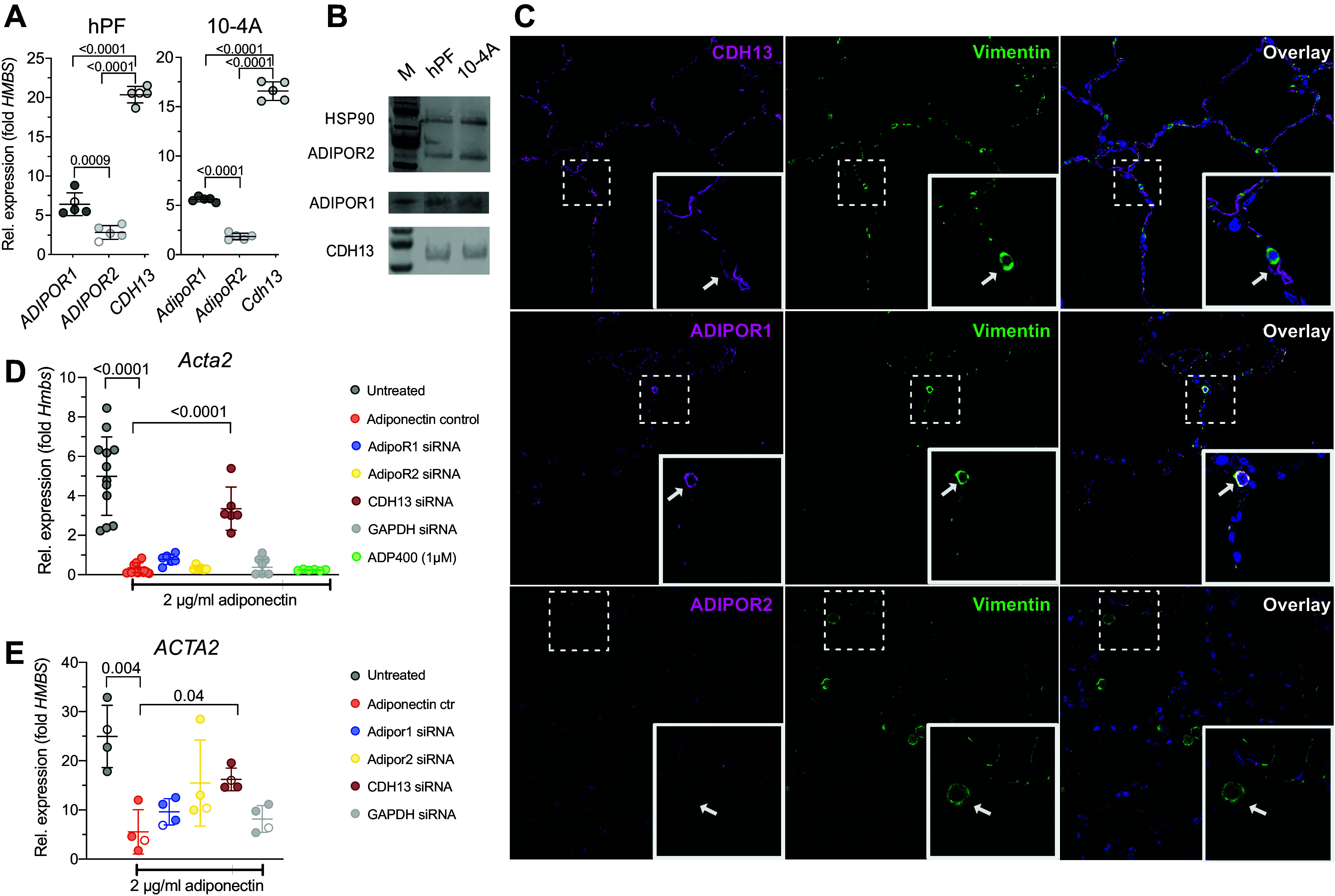
The antifibrotic effects of adiponectin depend on CDH13 expression in lung fibroblasts. *A*: real-time RT-PCR analysis of adiponectin receptor *ADIPOR1*/*Adipor1*, *ADIPOR2*/*Adipor2*, and *CDH13*/*Cdh13* transcript expression in primary human lung fibroblasts (hPFs) and 10-4A cells, respectively. Data are expressed as fold expression of housekeeping gene *HMBS*/*Hmbs*, *n* = 5 individual donors (hPF) or passages (10-4A). Significance was assessed by Kruskal–Wallis test with post hoc Dunn’s test. *B*: Western blots for ADIPOR1, ADIPOR2, and CDH13 expressed in hPF or 10-4A cells. HSP90 was used as loading control and for protein normalization. *C*: immunofluorescence staining for adiponectin receptors (ADIPOR1, ADIPOR2, and CDH13; purple) and vimentin (green) in human lung slices from healthy donors. Overlay images also show cell nuclei (blue). *Insets* show close-up of individual vimentin positive cell (arrow) from the dashed rectangle in the large image. Scale bars = 20 µm. *D* and *E*: real-time RT-PCR analysis of *Acta2* transcripts in 10-4A (*D*) and hPF (*E*) cultured for 7 days on plastic. Cells were transfected after 3 and 5 days with the indicated siRNAs (*D* and *E*) or treated with 1 μM of the ADIPOR1/ADIPOR2 specific inhibitor ADP400 (*D*) from *day 3* onward. 2 µg/mL rat (*D*) or human (*E*) adiponectin was added on *day 5*. *n* = 4–11 individual donors (hPF, ○ encode male donors) or passages (10-4A). Significance was assessed by Kruskal–Wallis test with post hoc Dunn’s test.

Next, we aimed at elucidating the signaling pathways underlying the antifibrotic effect of adiponectin. Adiponectin exerts its effects via binding to three known receptors. Canonical adiponectin receptors 1 and 2 (Adipor1, Adipor2) and T-cadherin (CDH13). All three receptors are expressed in primary hPFs and 10-4A cells (Fig. 4, *A* and *B*), with CDH13 being the highest expressed. Receptor expression was not affected by substrate rigidity (2 kPa, 16 kPa, plastic) or adiponectin treatment (Supplemental Fig. S4). Immuno-labeling of FFPE lung slices from human donor tissue confirmed expression of Adipor1 and CDH13 in vimentin-positive cells in the distal lung parenchyma, whereas no specific Adipor2 staining could be detected (Fig. 4*C*).

We performed siRNA knockdown (KD) of adiponectin receptors in 10-4A and HPF cells to further identify the role of individual receptors for the antifibrotic effect of adiponectin. siRNA treatment resulted in a 50%–95% reduction of individual receptor expression within the respective cells (Supplemental Fig. S5). KD of Adipor1 or Adipor2 did not alter the inhibitory effect of adiponectin on stiffness-induced *Acta2*/*ACTA2* expression (Fig. 4, *D* and *E*). In contrast, KD of CDH13 significantly reduced the antifibrotic effect of adiponectin (Fig. 4, *D* and *E*). These results suggest that the antifibrotic effect of adiponectin is dependent on CDH13, but not Adipor1 or Adipor2. In line, pharmacological inhibition of Adipor1 or Adipor2 by selective inhibitor APN400 did not affect adiponectin-mediated inhibition of *Acta2* expression in 10-4A cells grown on plastic (Fig. 4*D*).

### The Antifibrotic Effects of Adiponectin Are Mediated via p38 MAPK Signaling

**Figure 5.**
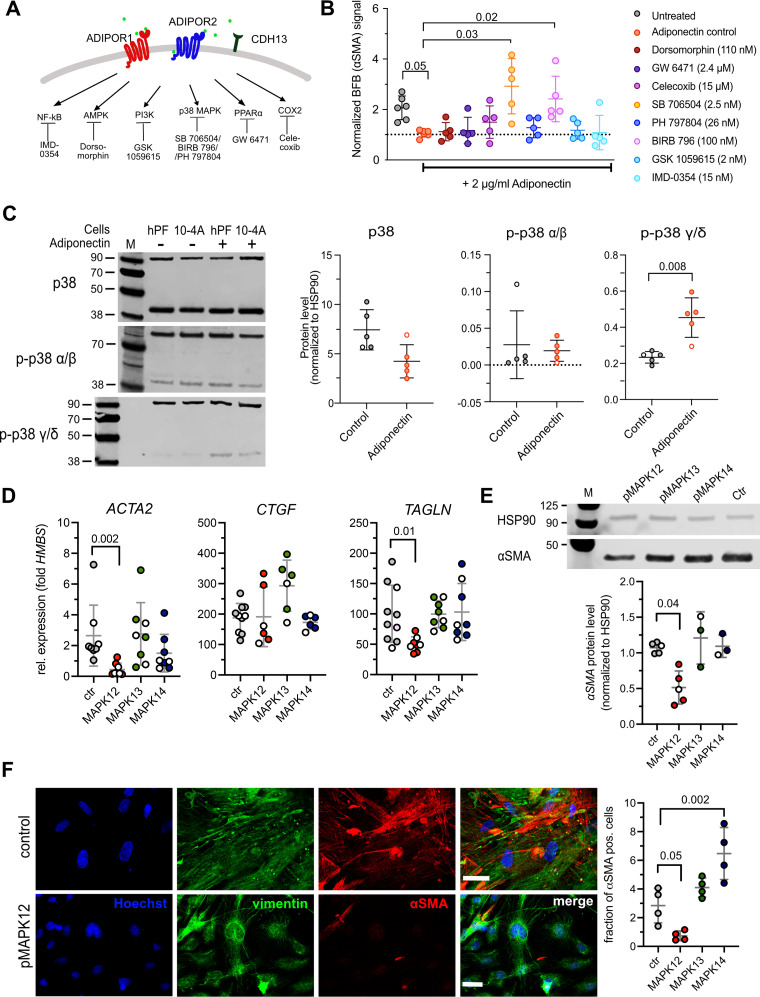
The antifibrotic effects of adiponectin are mediated via selective activation of the p38 mitogen-activated protein kinase gamma (p38 MAPK γ) isoform. *A*: schematic presentation of downstream signaling pathways of adiponectin receptor activation and the inhibitors thereof used in *B*. *B*: pharmacological screen to identify signaling pathways involved in the antifibrotic effect of adiponectin. 10-4A^BFP^ cells were seeded on plastic and treated with 2 µg/mL of rat adiponectin and the respective inhibitors for 7 days. The BFP signal was normalized to total cell number (determined by Calcein AM) and represents fibrotic activation (i.e. αSMA expression; BFP expression is under the control of the *Acta2* promoter). Data were obtained from six individual experiments. Significance was assessed by Kruskal–Wallis test with post hoc Dunn’s test. *C*, *left*: Western blots for p38, p38 α/β and p38 γ/δ expressed in hPF or 10-4A cells grown on stiff (plastic) substrate for 7 days in the absence (−) or prescence (+) of 2 µg/mL human (primary human lung fibroblasts, hpF) or rat (10-4A) adiponectin (*left*). HSP90 (*top band*, 90 kDa) was used as loading control and for protein normalization. *C*, *right*: densiometric analysis of protein expression in hPFs. Data were obtained from five independent experiments. ○ encode male donors. Significance was assessed by nonparametric Mann–Whitney *U* test. *D*–*F*: effect of selective expression of constitutive active forms of different p38 MAPK subunits (p38 MAPK phoshomimetics, pMAPK) on stiffness-induced fibroblast (hPF) activation. hPF activation was assessed on *day 7* on the transcript (*D*) and protein (*E*) level and analyzing the fraction of αSMA positive cells (*F*) in control cells and cells expressing the indicated pMAPK isoforms. HSP90 was used as loading control and for protein normalization in *E*. Data were obtained from 3–10 independent experiments. ○ encode male donors. Significance was assessed by nonparametric Mann–Whitney *U* test.

We next sought to identify specific signaling pathway(s) downstream of CDH13 that mediate the antifibrotic effects of adiponectin (Fig. 5*A*). We performed a pharmacological screen in 10-4A^BFP^ cells grown on plastic. BFP expression in these cells is linked to *Acta2* expression, and culture of 10-4A^BFP^ cells on stiff substrate results in increased BFP signals (23). Adiponectin (2 µg/mL) significantly reduced the BFP signal in 10-4A^BFP^ cells grown on stiff substrate (Fig. 5*B*). Selective inhibition of adiponectin-induced signal transduction pathways revealed that only inhibitors of p38 MAPK (SB706504 and BIRB 796) abrogated the effect of adiponectin on stiffness-mediated *Acta2* expression. Inhibition of all other adiponectin-induced signaling pathways did not alter the antifibrotic effect of adiponectin (Fig. 5*B*). Initial experiments indicated expression of all four p38 MAPK isoforms (α, β, γ, δ) in 10-4A cells and hPFs. The α isoform was the highest expressed isoform in both cells, whereas the β was hardly expressed in either cell type (Supplemental Fig. S6). Expression was not affected by substrate stiffness or adiponectin treatment (Supplemental Fig. S6). However, phosphorylation assays indicated specific activation (phosphorylation) of the γ/δ but not the α/β isoforms of p38 MAPK in hPFs treated with adiponectin (Fig. 5*C*). Selective expression of constitutive active forms of different p38 MAPK subunits (p38 MAPK phoshomimetics, pMAPK) further revealed that stiffness-dependent activation of hPFs, and 10-4A cells was selectively inhibited by active p38 MAPKγ (pMAPK12). Expression of pMAPK12 led to a significant reduction of *ACTA2/Acta2* and *TAGLN/Tagln* expression (Fig. 5*D* and Supplemental Fig. S7), reduced αSMA protein levels (Fig. 5*E*), and reduced the numbers of αSM-positive cells (Fig. 5*F*) in hPFs and 10-4A cells grown on stiff substrate, respectively.

In summary, our results suggest that the antifibrotic effect of adiponectin on stiffness-induced activation of lung fibroblasts depends on CDH13 expression and is mediated via selective activation of the p38 MAPKγ isoform.

### CDH13 Expression and p38 γ/δ Activation Are Reduced in Lungs from Patients with IPF

**Figure 6.**
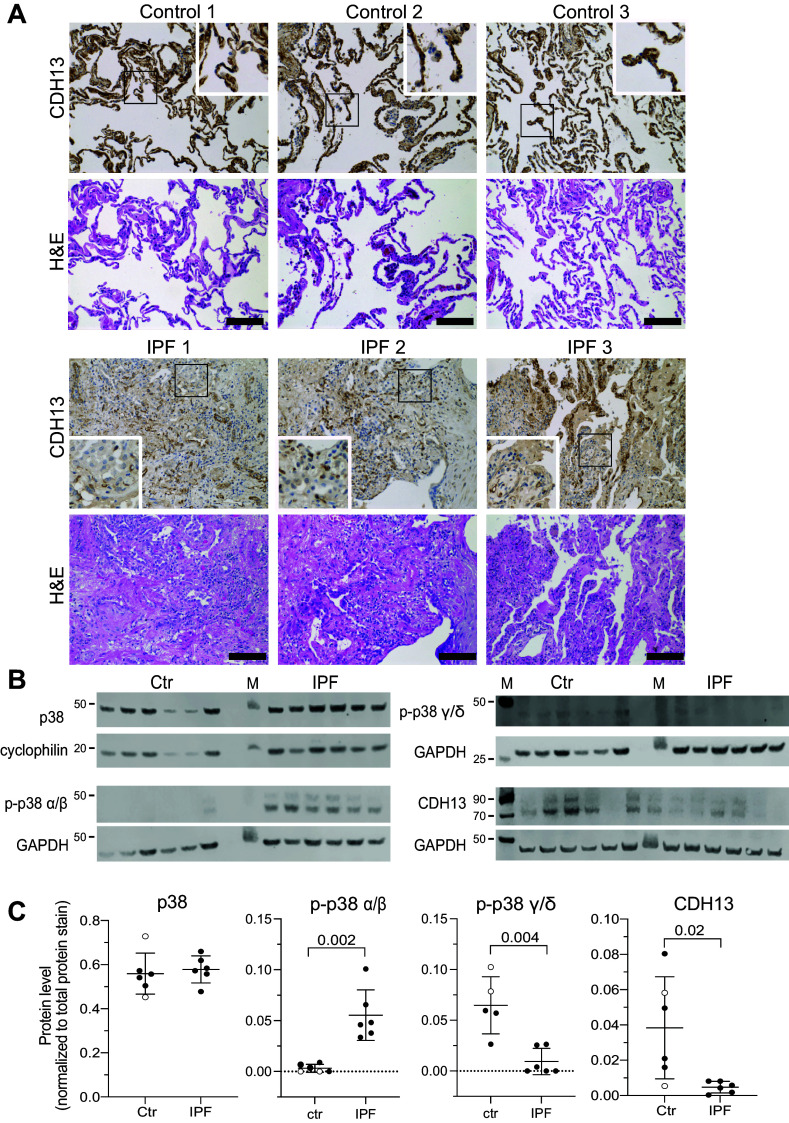
CDH13 expression and p38 γ/δ activation are reduced in lungs from patients with idiopathic pulmonary fibrosis (IPF). *A*: anti-CDH13 and hematoxylin and eosin (H&E) staining of healthy (control) and IPF lung tissue from three individual donors, respectively. *Insets* represent higher power images (×2.5) of the CDH13 staining within black rectangle in healthy and fibrotic lung regions. Scale bar = 100 µm. *B*: Western blots for p38, p38 α/β, p38 γ/δ, and CDH13 in human lung tissue from healthy donors and patients with IPF. GAPDH was used as loading control and for protein normalization. *C*: densiometric analysis of p38, phospho p38 α/β, phospho p38 γ/δ and CDH13 protein expression in healthy (control) and IPF lungs from six individual donors. ○ encode male donors. Significance was assessed by nonparametric Mann–Whitney *U* test.

Based on these findings we asked whether the adiponectin, CDH13, and p38 MAPK signaling axis might also be relevant in IPF. Previous studies have already reported reduced levels of adiponectin in lungs from patients with IPF compared with healthy control donors (20, 22). However, results were ambiguous with higher levels in patients with end-stage IPF (21). Adiponectin levels have been shown to depend on receptor expression; in particular, CDH13 constitutes a sink for adiponectin (36). Hence increased adiponectin levels at late stages of IPF could be a result of reduced CDH13 expression. We therefore analyzed CDH13 expression in distal lung tissue from healthy donors and patients with IPF. Immunohistochemistry and Western blot confirmed a significant, almost complete reduction of CDH13 in the distal lung of patients with IPF, with little CDH13-positive fibroblasts (Fig. 6 and Supplemental Fig. S8). In line, data from recent RNAseq and microarray studies (GSE134692, GSE10667, GSE31962, GSE173355, GSE32539) (37–41) indicate that expression of all adiponectin receptors is downregulated in fibroblasts isolated from IPF donor tissue when compared with fibroblasts isolated from healthy control tissues. CDH13, in particular, is significantly downregulated across all datasets (Supplemental Fig. S9). In addition, we found that activation (phosphorylation) of the γ/δ isoforms of p38 MAPK was significantly downregulated in human lung tissue from patients with IPF. Interestingly, phosphorylation of the p38 α/β isoform was significantly increased in human IPF lung tissue (Fig. 6).

**Figure 7.**
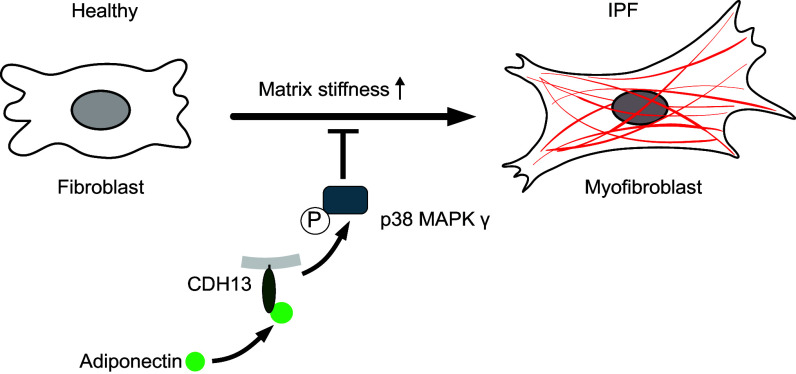
Proposed model of the adiponectin-induced signaling cascade that inhibits stiffness-dependent profibrotic fibroblast activation. Adiponectin attenuates mechanically induced fibroblast activation via binding to CDH13 and downstream activation (phosphorylation) of the γ isoform of p38 MAPK. Specific targeting of this adiponectin-induced signaling cascade may provide therapeutic benefits in idiopathic pulmonary fibrosis (IPF).

These data suggest that reduced CDH13 expression and reduced activation of the p38 MAPKγ may be linked to IPF. Together with our results from the in vitro experiments, this is likely the result of a reduced inhibition of stiffness-dependent fibroblast activation (Fig. 7).

## DISCUSSION

To date, nintedanib and pirfenidone are the only approved drugs for treatment of IPF (42, 43). Both drugs slow down disease progression, but do not halt the disease, offering limited benefits in reducing symptoms and prolonging life (44). Identifying additional targets for drug development is urgently needed to fight IPF. Nintedanib and pirfenidone were discovered when the exact pathophysiology of IPF was still elusive. Extensive research has now identified several mechanisms underlying the onset and progression of the disease (45–47). It has become evident that persistent activation of myofibroblasts and excessive deposition of ECM in response to increased matrix stiffness of the fibrotic tissue is a central driver of disease progression (4, 6–11). Within this study we set out to identify novel signaling pathways underlying this mechano-signaling induced fibroblast activation. As we identified signaling pathways were altered in response to increased substrate stiffness, we compared fibroblasts grown on a physiologically soft (3 kPa) and unphysiologically rigid (plastic, GPa) substrates. However, differentially expressed genes can change significantly between a substrate with rigidity found in lung tissue of patients with IPF (∼15–100 kPa) and plastic (4). This might affect gene and pathway enrichment, hence future experiments determining gene and pathway expression on substrates in the 15–100 kPa might reveal additional targets not identified within our study.

Within our study, we found that adipocytokine signaling, specifically adiponectin signaling via CDH13, exerts antifibrotic effects in pulmonary fibroblasts and inhibits mechanically induced activation of lung fibroblast. This is in line with several previous reports of adiponectin serving as antifibrotic mediator. Adiponectin has been found to exert antifibrotic activity in various organs including liver, skin, heart, kidney, and the lung (16–18). In the lung, adiponectin protected against paraquat-induced pulmonary fibrosis by decreasing fibroblast activation (19) and significantly attenuated bleomycin-induced body weight loss, alveolar destruction, and collagen fiber accumulation in mice (48). Wang et al. also found decreased expression of α-SMA and collagen I in a lung fibroblast cell line following treatment with profibrotic mediator TGF-β1. In this study, the antifibrotic effects of APN were possibly mediated via inhibiting the NF-κB signaling pathway (48). Our data suggest a central role for adiponectin in inducing CDH13-dependent phosphorylation and activation of γ isoforms of p38 MAPK. Differences in the underlying intracellular signal transduction that elicit the antifibrotic impact of adiponectin on lung fibroblasts might result from the difference in fibroblast activation (i.e., TGF-β1 vs. substrate stiffness). However, such broad antifibrotic effect further strengthen the evidence that adiponectin might be a potential antifibrotic in IPF (48).

The potential relevance of our findings for IPF is also supported by previous RNAseq and microarray studies (37–41). These suggest that the expression of adiponectin receptors, in particular CDH13, is reduced in patients with IPF. Whether the reduction of CDH13 expression is a consequence of reduced adiponectin levels or cellular differentiation needs to be determined. In our short-term experiments (up to 14 days), we did not see a significant change in CDH13 expression. However, levels of CDH13 protein were reduced in adiponectin-deficient mice and were increased by adiponectin (49). It is therefore tempting to speculate that adiponectin signaling through CDH13 support the expression of CDH13 via a positive feedback loop mechanism. Interestingly, there is also a reciprocal relationship, with plasma adiponectin levels being increased in CDH13 knockout animals, likely due to reduced tissue binding and sequestration of adiponectin (49). Accordingly, altered expression of CDH13 under pathological conditions could then lead to changes in the amounts of circulating and tissue-localized adiponectin (50). This could explain recent observations that serum adiponectin levels were decreased in patients with IPF at diagnosis but increased in patients with IPF with acute exacerbations (21). Similarly, a recent study also noted adiponectin signals in thin alveolar septae of healthy donors, but not in patients with IPF (20). Yet, it is still to be determined whether adiponectin levels in serum, BAL, or tissue are relevant in IPF. Adiponectin levels in BAL are orders of magnitude lower than in serum (51).

The detailed signaling cascade linking adiponectin binding to CDH13 and activation of p38 MAPK is still elusive. p38 MAPK signaling is highly regulated and activation involves multiple dedicated kinases to integrate upstream inputs (52). Moreover, it is still enigmatic, how CDH13 initiates the intracellular signaling cascade. CDH13 is anchored to the plasma membrane via a glycosylphosphatidylinositol (GPI)-anchor and lacks transmembrane and cytoplasmic signaling domains. This implies the interaction with a membrane adapter protein to relay the signal into the cell. It has been proposed that integrin-linked kinase (53), which has been found to mediate mechanical stress-induced signals (54, 55), is an essential mediator of CDH13 signals into inward signal transmission (56). It has also been proposed that CDH13 is located in caveolae, caveolin-rich plasma membrane domains (57). Caveolae sequester intracellular signal molecules (58), mediate mechano-adaption to different pathological processes (59) including responses to substrate stiffness (60), and have been linked to driving mechanically induced fibrosis (61). Yet, the jury is still out on the molecular mechanisms of CDH13 transmembrane signaling and it will be interesting to see whether future studies can reveal a specific mechanism for the CDH13-induced antifibrotic effect in fibroblasts.

The antifibrotic effects of p38 MAPK γ-isoform activation are interesting. So far, p38 MAPK signaling has been mainly associated with profibrotic responses in various organs (62, 63) including the lung (64) and inhibition of p38 MAPK signaling-induced antifibrotic effects. In kidney fibroblasts and a pterygium of the eye, blockade of p38 MAPK signaling inhibited TGF-β1-induced αSMA and collagen expression (65, 66). These results may seem contradictory to our findings, but may result from the use of non-isoform-specific inhibition of p38 MAPK. Most studies used either pharmacological tools specifically acting on the α isoform of p38 MAPK or pan-p38 MAPK inhibitors. Our data therefore highlight the importance of isoform specific modulation of p38 MAPK activation. Isoform specific expression of pMAPK identified that only activation of the γ isoform serves as specific inhibitor of stiffness-induced fibroblast activation. Interestingly, transcriptomics data obtained in the bleomycin mouse model also found a reduction in the expression of the γ isoform (MAPK12) (67). Although, we could not directly test whether activation of p38 MAPKγ attenuates fibroblast activation and fibrotic remodeling of the lung in IPF donors, we found that activation of the α/β isoforms was increased whereas activation of the γ/δ isoforms was decreased in distal lung tissue from patients with IPF. Whether the antifibrotic changes through pMAPK12 are achieved via *1*) direct activation of downstream transcription factors, *2*) master regulators of fibroblast phenotype, or *3*) classical mechano-signaling pathways needs to be determined and is currently under investigation.

Our findings are also interesting in the context of a recent study that found that pirfenidone, one of the two approved drugs for treatment of IPF, binds to and inhibits p38 MAPKγ (68). In this study, pirfenidone inhibited cell proliferation and protected against chemically induced formation of liver tumors. This was attributed to specific inhibition of the cyclin-dependent kinase behavior of p38 MAPKγ. We did not observe any effects of adiponectin treatment, and hence activation of p38 MAPKγ, on fibroblast proliferation, only on fibroblast activation. Still, whether pirfenidone could potentially also have adverse effects in the treatment of IPF by inhibiting p38 MAPKγ needs to be tested.

Overall, our findings strongly suggest an inverse relationship between adipocytokine signaling and IPF. However, based on our findings that CDH13 is downregulated in patients with IPF, we suggest that administration of adiponectin receptor agonists (69) might not be a promising treatment option. This is supported by a recent study reporting that metformin treatment, which increases adiponectin serum levels (70), had no positive effect in patients with IPF (71), possibly due to reduced adiponectin receptor levels (CDH13). Therefore, treatment aiming at increasing CDH13 expression (in combination with administration of adiponectin receptor agonists) and/or selectively targeting the downstream signaling cascade, i.e., activation of the p38 MAPK γ isoforms, could be promising therapeutic targets in the treatment of IPF.

## DATA AVAILABILITY

Data will be made available upon reasonable request.

## SUPPLEMENTAL MATERIAL

10.25833/g4qt-3356 Supplemental Figs. S1–S9: https://doi.org/10.25833/g4qt-3356.

## GRANTS

This work was funded by the Boehringer Ingelheim Ulm University BioCenter (BIU 2.0) and the Deutsche Forschungsgemeinschaft (DFG, German Research Foundation)—Projektnummer(n): 278012962 (GRK “Pulmosens”), 251293561 (SFB1149), and 175083951 (to M.F.).

## DISCLOSURES

No conflicts of interest, financial or otherwise, are declared by the authors.

## AUTHOR CONTRIBUTIONS

J.N. and M.F. conceived and designed research; J.N., S.K., A.S., T.S., K.Q., C.R., and A.G. performed experiments; J.N., S.K., A.S., A.G., and K.Q. analyzed data; J.N., W.S.-W., A.G., and M.F. interpreted results of experiments; J.N. and M.F. prepared figures; J.N. and M.F. drafted manuscript; J.N., W.S.-W., and M.F. edited and revised manuscript; J.N., W.S.-W., S.K., A.S., A.G., T.S., K.Q., K.C.E.K., C.R., A.G., and M.F. approved final version of manuscript.
